# The Effects of Exergames on Fundamental Movement Skills in Childhood: A Systematic Review and Meta-analysis

**DOI:** 10.1186/s40798-026-01081-2

**Published:** 2026-09-20

**Authors:** Oscar O’Brien, Connah Kendrick, Ben Ives, Moi Hoon Yap, John Henry

**Affiliations:** 1 Faculty of Science and Engineering, Department of Computing and Mathematics, Manchester Metropolitan University, Dalton Building, Chester Street, Manchester, M1 5GD United Kingdom; 2 Faculty of Science and Engineering, Department of Sport and Exercise Sciences, Manchester Metropolitan University, Dalton Building, Chester Street, Manchester, M1 5GD United Kingdom

**Keywords:** Exergames, Exergamoids, Fundamental movement skills, Physical activity, Childhood

## Abstract

**Background:**

Physical inactivity is a public health crisis that is influenced by practical barriers in childhood. We define a category of interventions that can reduce barriers, with exergames being one example. There is mixed evidence on fundamental movement skills effects, due to variability in experimental outcomes, that may be limiting usage. This meta-analysis identifies the source of that variability to determine the effect of exergames on fundamental movement skills.

**Methods:**

To provide a comprehensive evidence base, we systematically surveyed 10 research databases / search engines (PubMed, APA PsycINFO, SPORTDiscus, IEEE Xplore, ACM Digital Library, Scopus, Proquest, Web of Science, ScienceDirect, and Google Scholar) to identify studies of the post-intervention effects of exergames upon children's fundamental movement skills, published between Jan 2000 and Dec 2025. Records were identified through boolean search terms and then manual reviews of abstracts and full text. We used set theory and order theory, in an approach we call systematic hypothesis testing, to explore all explanations for fundamental movement skills variability.

**Results:**

In total, 17 statistically significant comparisons from 12 studies were identified. Comparisons between groups involved variance in coaching levels, activity structure levels, and whether the activity was an exergame or not. This study demonstrates that variability in fundamental movement skills effects is explained by coaching and activity structure, rather than exergame status. There was no evidence of a differential effect for skill components.

**Conclusions:**

Low-barrier exergames, as defined in our taxonomy of exergame types, are just as effective as traditional activity for fundamental movement skills adaptation. They should be freely chosen by teachers and parents, to overcome the practical challenges that are spiralling into physically inactive lifestyles. This study provides a taxonomy and model of statistically significant effects that future research should use to manage the methodological variability that has made building upon previous findings challenging.

## Key Points

- This meta-analysis provides definitive evidence that low-barrier exergames are just as effective as traditional activity for fundamental movement skill adaptation, in experimental conditions. The implications are that if practitioners are able to make use of exergames in practice, selecting activities based on needs, structuring activities, and coaching them like a traditional activity, then this should be considered a legitimate physical education activity.
- Coaching during low-barrier exergame play enhances fundamental movement skills, with no significant difference in overall performance levels when compared with coaching during a traditional activity. More research is needed on the fundamental movement skills effects of other forms of exergamoids, exergames with simulated coaching, and exergames with co-teaching systems.
- The conceptual model we develop explains how exergamoids may offer unique benefits as a community-based lifestyle intervention that complements existing strategies. Exergamoids can motivate fundamental movement skill acquisition in school and overcome practical barriers to physical activity in the home environment. By creating an activity opportunity that spans both settings, movement skills learned in school have an opportunity to translate to a pattern of engagement in physical activity at home.

## Background

More than 25% of adults [1] and 80% of adolescents [2] are not meeting recommended amounts of physical activity (PA) for their age-range. Low PA is a global crisis, as active lifestyles have been linked to delaying the onset of 40 chronic conditions/diseases [3]. [4] Estimated that physical inactivity causes almost 50 million new cases of non-communicable diseases globally each year. With no change in activity trends [4] estimates the cost of treating these non-communicable diseases to around US$ 300 billion between 2020 and 2030. An effective juncture to address inactivity is in childhood, where a spiral of behaviour can precipitate an inactive lifestyle [5, 6]. Put forth a conceptual model of the relationship between motor skills competence and engagement in physical activity to illustrate that motor skill competence is a primary mechanism that promotes active lifestyles. The model illustrates a negative spiral, where low PA inhibits motor skills and inter-related variables, which in turn inhibit PA even further [6]. An implication of the model is that PA is expected to translate to benefits in motor skills, to in turn fully trigger a positive spiral that improves participation in PA. Considering that the motor skills required to access PA can vary widely depending on the type of PA, the breadth of motor skills developed through PA in childhood is an important consideration. Fundamental movement skills (FMS) are basic movement patterns that can be refined into the specialised movements required for participation in more complex physical activities [7]. Locomotion such as running and jumping, manipulating objects such as striking with a bat and catching a ball, and maintaining stability such as the beam walk and 1-foot balance are examples of FMS [7]. Developing a broad range of FMS through diverse PA in childhood may build a more robust foundation for lifelong health than focusing on PA alone.

Physical education and school sport (PES), and physically active play at home are childhood settings, where interventions to promote PA interact. When viewed under Stodden et al.'s model [6], PES would have the long term objective of developing FMS in childhood to enable engagement in PA in later life. Time allocated to PE, typically 60.6−130.6 min per week [8], falls short of the minimum activity guidelines put forward by the [9] of at least an average of 60 min per day of moderate to vigorous activity. Home-based PA could be considered ‘homework’ of PES sessions, where children build their fitness levels and practice the movement skills that have been introduced in PES. This PA ‘homework’ is essential for PES to function as a public health intervention, given limited contact time in PES. Trends in activity indicate this is not happening for the majority of children. A factor behind current inactivity levels is considered to be excessive screen time, displacing time that could otherwise be spent on physical activities [10]. Concerns around screen time have led to guidelines, exemplified by the [9], recommending young people aged 5–17 limit sedentary screen time. Limiting screen time is not easily achievable due to: increased family conflict [11], increased demands on parental time and energy that may be occupied with work or chores [11], financial constraints, such as not living near safe outdoor areas or owning a big garden [11], and no incentive to switch to exercise instead of alternate sedentary activities [11]. Implementation challenges may explain why there was no significant evidence of interventions involving screen time limits achieving an actual reduction in screen time for children [12, 13]. If appealing home-based PA was more readily available, and PES curriculum was planned to enable participation in these activities, then sedentary screen-time limits and PES could be more effective in improving participation in PA.

### Exergamoids

**Fig. 1:**
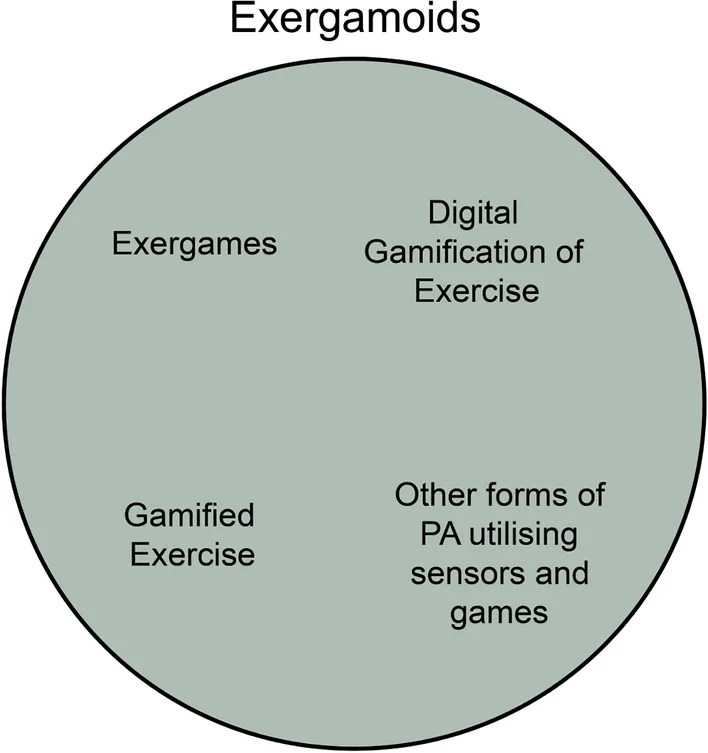
Euler diagram of exergamoids. Exergames, digital gamification of exercise, gamified exercise, and other forms of PA utilising sensors and games are examples of exergamoids

**Fig. 2:**
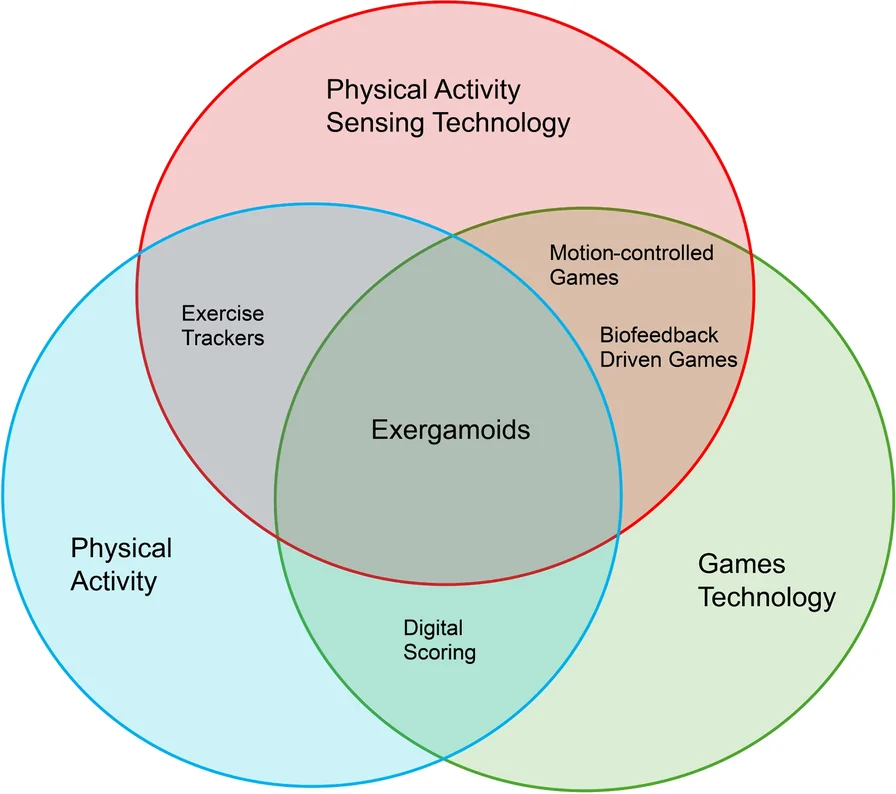
Venn diagram of exergamoids. We define exergamoids as physical activities that involve sensors of physical activity controlling a games technology component of the experience

An alternate approach to promote active lifestyles is to make use of the appeal of digital games to motivate FMS acquisition in school and overcome practical barriers to autonomy by facilitating PA in their home environment. Games technology can integrate with PA through a loop:

1. Digital tracking of PA is used as an input to a games technology component.
2. The games technology component produces output that augments the physically active experience.

There are different methods by which the mechanism of PA input and game system output can be applied to create physically active experiences. Games technology can integrate PA in the format of a complete game. A complete game involving PA would be considered an exergame. Exergames are defined as games, where players develop their health in various ways as they play [14]. Games technology can integrate PA in a format that would not be categorised as an exergame, because the digital component is not a complete game. Gamification is defined by [15, p. 10] as ‘the use of game design elements in non-game contexts’. Progression systems from games could augment step counters with a level system to make running more engaging. There are some experiences that do not fit an existing games technology category. A larger physically active experience can be created by combining exergames with traditional circuit training, as seen in the study by [16]. While the requirements of applications for games, digital gamification, game-based approaches are different, applying them in a PA context creates commonalities between them. We propose the term 'exergamoids' to organise knowledge and facilitate transfer of findings. We define exergamoids as physical activities that involve sensors of physical activity controlling a games technology component of the experience (see Figs. 1 and 2). Exergamoids share methods that can be employed to detect successful movement skill performances, reward users, and solve contextual game design problems. Exergamoids have shared pathways of real world effect, such as game-based motivation of physical movement creating health outcomes.

### The Need for Meta-analysis of the Fundamental Movement Skills Effects of Exergamoids

Exergamoids could facilitate PA at home. As exergamoids are self-running they could enable children to be playing different games simultaneously, optimising motivation. The self-refereeing nature of digital scoring and the virtual equipment reduce logistical burdens on the teacher. The paediatric inactivity triad indicates that exergamoids would need to be vehicles for PA, physical literacy and strength training, to make efficient use of limited time in school settings, where efficient traditional activities are available.

The effectiveness of exergamoids for PA and strength training can be determined, by practitioners, through comparing the movements and equipment involved in the exergamoid with traditional activity. Methods to determine the effectiveness of exergamoids for FMS adaptation remain inconclusive. [17] Found mixed evidence on the effects of active video games upon locomotor and object manipulation skills. [17] Could not conduct a meta-analysis due to heterogeneity. A meta-analysis by [18] found mixed neutral and positive motor skill effects, indicating a potentially positive effect. [18] Observed a trend of enhanced effects related to balance and speculated that this was due to specificity. Parents generally believed that exergames cannot support FMS acquisition [19]. The continuing mixed evidence on the effectiveness of exergamoids combined with initial scepticism may be limiting confidence in using exergamoids for health benefits across childhood settings. Our meta-analysis aims to examine if the mixed evidence on the effects of exergamoids on FMS can be explained by methodological variability, to conclusively identify the effects of exergamoids, and to determine the factors that influenced results.

## Methods

### Research Questions

There is mixed evidence on the effectiveness of exergamoids for improving FMS. As a result of uncertainty over FMS effects, it is not known if the use exergamoids in PES would contribute to increasing PA in later life in the same way traditional activities do, when viewed in the context of Stodden et al.'s [6] model of the relationship between motor competence and PA. We hypothesise that mixed evidence is caused by variance in methodology and that controlling for methodological variability could clarify the FMS effects of exergamoids. No existing meta-analysis has systematically explored the methodological variables and the types of effect they have on FMS: positive, negative, neutral, or unknown. Our meta-analysis addresses this gap in existing knowledge by determining the cause of variance in exergamoid effects on FMS through the following questions:

- RQ1: What methodological variables exist amongst research into the use of exergamoids to improve children’s FMS?
- RQ2: Can the variance in FMS effects be associated with variances in the methodology?
- RQ3: What type of effect does each methodological variable have on FMS?

Our meta-analysis clarifies the effects of exergamoids on FMS, and identifies future research directions. Conclusions are then drawn about the potential utility of exergamoids for public health.

### Eligibility Criteria

Population, Intervention, Comparison, Outcome (PICO) criteria, as suggested by [20], were used to systematically specify the scope of the study, the inclusion criteria, and search terms.

- *Population*: Studies must focus on childhood: ages 4–12 to be included in our meta-analysis. Our meta-analysis focuses upon the FMS effects of exergamoids for the entire population of children, and in all settings. Studies involving a specialised population, where all participants had a specific medical condition or cognitive need, were not explored within our meta-analysis.
- *Intervention*: Studies must involve the use of an exergamoid.
- *Comparison*: No comparison group criteria were chosen in order to include literature on exergamoids in all childhood settings.
- *Outcomes*: Studies must measure an effect on movement skills that contributes to engagement in physical activity.

In addition to PICO, we used the following criteria: the article must not be a review article, must be peer reviewed, and an English version of the full text must be available. For inclusion in the meta-analysis there must be a post-test FMS comparison with an assessment of statistical significance, and the chosen experimental variables must appear at least twice within the corpus to enable comparison. As the purpose of this review was to test if methodological variables can fully explain variability in FMS effects, regardless of methodological quality or risk of bias, no quality assessment was conducted. Because our analysis is of ordinal results rather than cardinal effect sizes, pooled effects are not being estimated which reduces sensitivity to bias. In other types of reviews, where pooled effects are not being estimated, such as scoping reviews, risk of bias assessment is not necessary [21].

### Search Strategy

To ensure good coverage of extant literature, we surveyed 10 research databases/search engines, including domain specific databases relating to the fields of health, computer science, sport science, psychology and multi-disciplinary databases/search engines: specifically PubMed, APA PsycINFO, SPORTDiscus, IEEE Xplore, ACM Digital Library, Scopus, Proquest, Web of Science, ScienceDirect, and Google Scholar. Search terms were developed from the PICO criteria (see Sect. Eligibility Criteria).

- *Population*: Context specific synonyms for children were not used as they could introduce a selection bias, which is inappropriate for a population term. For example, using ‘pupil’ as a search term will be more likely to match experiments performed in school settings. Only synonyms for children were used, and are listed in Table 1.
- *Intervention*: Games platform terms such as “Wii” or “Kinect”, or specific games such as “Dance Dance Revolution” can identify exergames research; however, these terms were not used in this meta-analysis due to introducing a potential selection bias towards commercial games and hardware. Instead, we chose broader terms such as “exergame” “virtual reality” or “gamification”, and their synonyms, which are listed in Table 1.
- *Comparison*: Defining keywords for all types of comparisons was not necessary as omission of the term would match all possible comparisons in a more reliably exhaustive way.
- *Outcomes*: Studies that measure an outcome relevant to fundamental movement skills were chosen over gross motor skills to focus on the skills that contribute to engagement in physical activity. To identify these skills, all papers relating to motor skills were found, and then a manual review of the abstract, and then full text if needed, determined if the motor skill is a valid fundamental or specialised movement skill. The search terms covered motor skills, fundamental movement skills and their synonyms, and are listed in Table 1.

A total of 2882 records were retrieved to create the ineligibility identification keywords. We developed ineligibility identification keywords by searching with the eligibility identification keywords and reviewing abstracts against the PICO criteria. Ineligibility identification keywords were extracted from the abstract through identification of the population, intervention or outcome that was out of scope for this study. Ineligibility keywords are listed in Table 1. We chose not to use ‘obesity’ as an ineligibility identification keyword due to over a quarter of exertion game articles making reference to it as part of discussion [22]. Exercise-related health conditions were not used as exclusion keywords, so studies of health effects on non-specialised populations could be passed on to the next stage of the study selection process. We excluded studies, where it was evident all participants had an exercise-related health condition during the abstract and full-text screening stages.

Abstracts for 545 articles that matched the search string were then downloaded. Some search engines did not support the required amount of AND/OR keywords to make the full search string involving all the synonyms, which would limit the number of synonyms that could be used. Where needed, multiple searches were run to cover all the possible combinations of the synonyms and the results were compiled manually. The searches identified terms in the title, abstract, and keywords. Where one of these elements was not available on a database or search engine the remaining elements were used. Any modifications to the search due to limitations are listed in the supplementary materials for each database/search engine.

**Table 1:** Search string used to identify studies via research databases/search engines

Keyword Type	Complete search string
Intervention Eligibility Identification	((exergame OR exergames OR exergaming OR “active video game" OR “exercise game" OR “exercise games" OR “augmented reality" OR “virtual reality") OR (“gamification" OR “gamify" OR “gamified" OR “gameful" OR (“pervasive" AND “game") OR “game elements" OR “game mechanics" OR “funware"))
Outcome Eligibility Identification	AND (“motor skills" OR “motor skill" OR “motor competency" OR “motor competence" OR “motor development" OR “motor learning" OR “motor ability" OR “FMS" OR “movement skill" OR “movement quality" OR “movement control" OR “movement performance")
Population Eligibility Identification	AND (child OR children OR young OR youth OR childhood)
Ineligibility Identification	NOT (rehabilitation OR therapy OR disability OR ADHD OR autism OR “down syndrome" OR disorder OR “medical skills" OR surgery OR surgical OR dentistry OR “fine motor" OR “young adult" OR “young adults")

### Screening and Eligibility Review

Some of the total of 545 abstracts were gathered by downloading a .ris file where provided by the database. Where a .ris file was not available, PDFs were downloaded and bibliographic information was constructed with the assistance of Mendeley. Bibliographic information was then checked against the PDF and then exported as a .ris file. There were 45 studies that were eliminated at this stage due to unavailability of an English full text.

A total of 500 bibliographic entries contained within the .ris files were then uploaded to a systematic review management platform.^1^ There were 216 duplicates, which were removed in two stages. In the first stage of duplicate removal, the software flagged potential duplicates for human review and their deletion from the dataset. In the second stage, the bibliographic information was then reviewed manually to remove any additional duplicates that had not been flagged by the software.

**Fig. 3:**
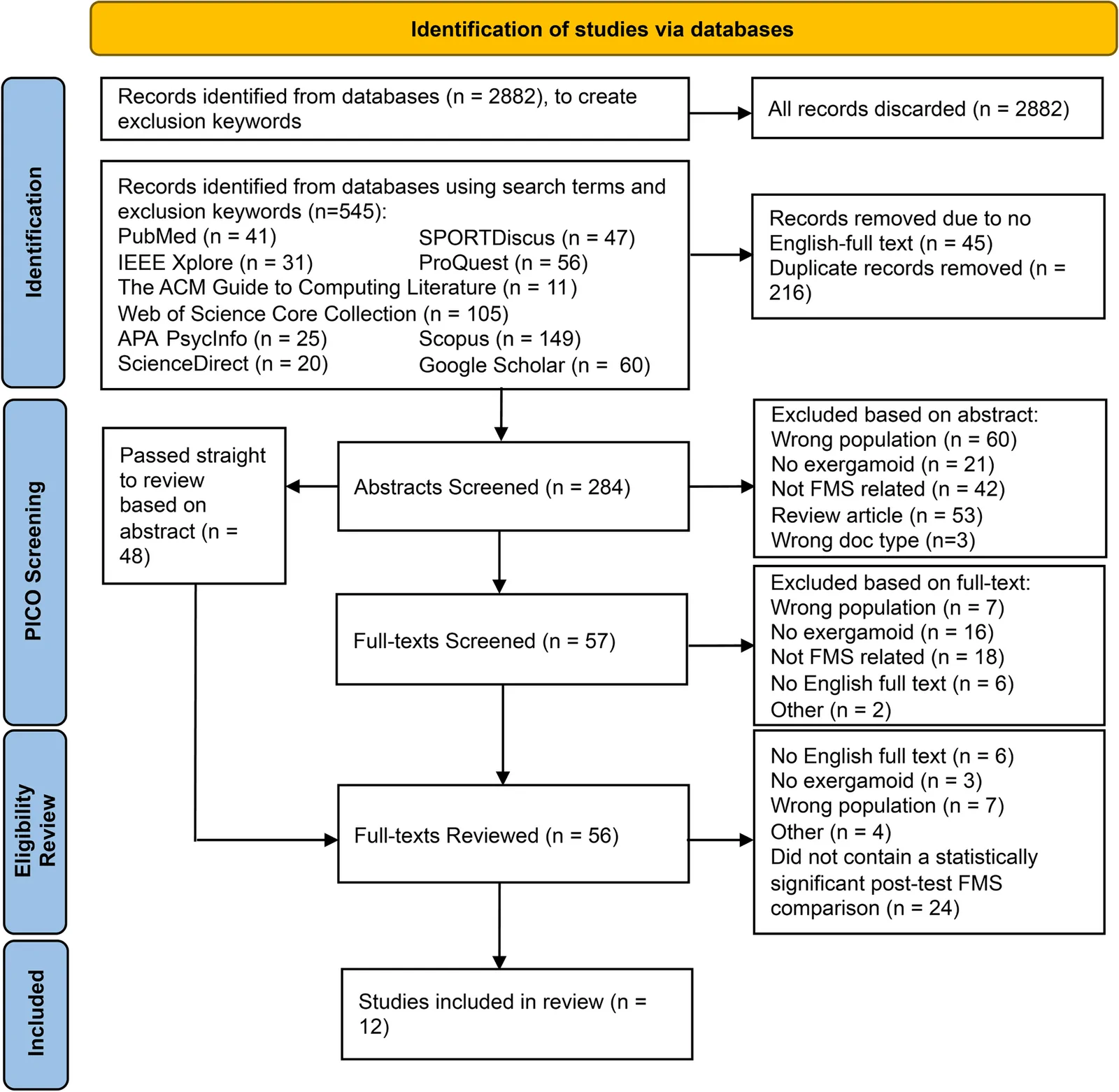
PRISMA flow diagram of the search results and studies identified for inclusion. *FMS* Fundamental Movement Skills

A total of 284 abstracts were screened and the papers were either passed to the full-text review stage or excluded, where this was possible to determine from the abstract using our PICO criteria (see Sect. Eligibility Criteria). There were 48 papers that were immediately passed to the full-text review stage and 179 that were excluded based on the abstract. The remaining 57 studies were passed to the full-text screening stage. The full text was screened using our PICO criteria, resulting in 8 more studies being passed to the review stage and 49 more being excluded. In total, 56 articles were passed to the full-text review stage. During the full-text review, we assessed if the study contained a statistically significant post-test FMS comparison and continued to match our PICO criteria, as shown in Fig. 3. In total, 12 studies were identified for our meta-analysis. Some studies involved multiple experimental groups and made multiple comparisons. In total, there were 17 comparisons between 28 intervention groups that were reported as statistically significant.

### Content Analysis and Classification Scheme

To evaluate the differential effects across FMS transfer, we identified papers, where the effects of the exergame activity were measured in a skills assessment outside of the game. Assessments of FMS transfers were categorised according to the skill aspects involved in the movement during the assessment. Several taxonomies for classifying movements skills exist [7]. Temporal, Environmental, and Functional skill taxonomies were selected, due to their overlap with different types of challenges sensors may face. The temporal taxonomy classifies how the movement occurs over time: a single event with a clearly defined start and end, being performed in rapid succession, or being performed repeatedly for an arbitrary length of time [7]. The environmental taxonomy classifies the context in which the movement occurs: an unpredictable and constantly changing environment, or a stable unchanging environment [7]. The functional taxonomy classifies the purpose of the movement: to gain or maintain balance, to transport from one point to another, or to impart force or receive force from an object [7]. Skill categorisations were manually verified by all authors according to the definitions in [7]. Some papers in our corpus specified the movements in their FMS transfer assessment indirectly through reference to a movement skills assessment framework, such as the Test of Gross Motor Development—3rd edition (TGMD-3). The skill aspects that were involved in the transfer scenario movements were determined from the referenced source in these cases.

To determine which types of exergamoids are more frequently utilised in children’s motor competency research, the methodology section of each study was reviewed to identify the games and hardware platforms that were used. There were 22 unique games, counting titles such as Wii Sports as a single game, and 4 different hardware platforms. Games were categorised as commercial, research, or not specified depending on the author’s indication. Where the game was not specified as commercial or researcher made, additional searches were performed. An internet search was used to identify commercial games through evidence of sale before the publication of the study of the game, such as store listings, promotional material or published reviews. The 10 research databases/search engines, as mentioned in Sect. Search Strategy, were searched for publications involving the game title, which were then reviewed for author indications to establish if the game was made for research purposes. Our protocol was that any games that were still unspecified were to be clarified through contact with the authors of the papers, but this was not necessary. The type of exergamoid was identified through descriptions of the game in the paper. Criteria for the potential categories were defined using standardised definitions, which are provided in the supplementary materials. In the game classification, categorisations were manually validated by all authors.

Methodological variables were identified based on descriptions of differences between control and intervention groups made within the study. We collected a short description of the relevant methodological details from the paper. After all descriptions were gathered, the methodological variables of the corpus were identified and clustered based on reported methods. The methodological variables that were identified are listed in Sect. What Methodological Variables Exist Amongst Research into the use of Exergamoids to Improve Children’s FMS? (RQ1). Categories were created to fit the data for each methodological variable, and all experimental groups were classified in those variables. Classifications were chosen to preserve any reported variance in methodology between groups that have been directly compared in papers. To enable comparison through meta-analysis, categories were ranked to determine structure level or coaching level. Where more methodological detail was available in the paper, categorisations were made in more detail. The categorisations created through textual meta-analysis and clustering align with conventional definitions.

**Fig. 4:**
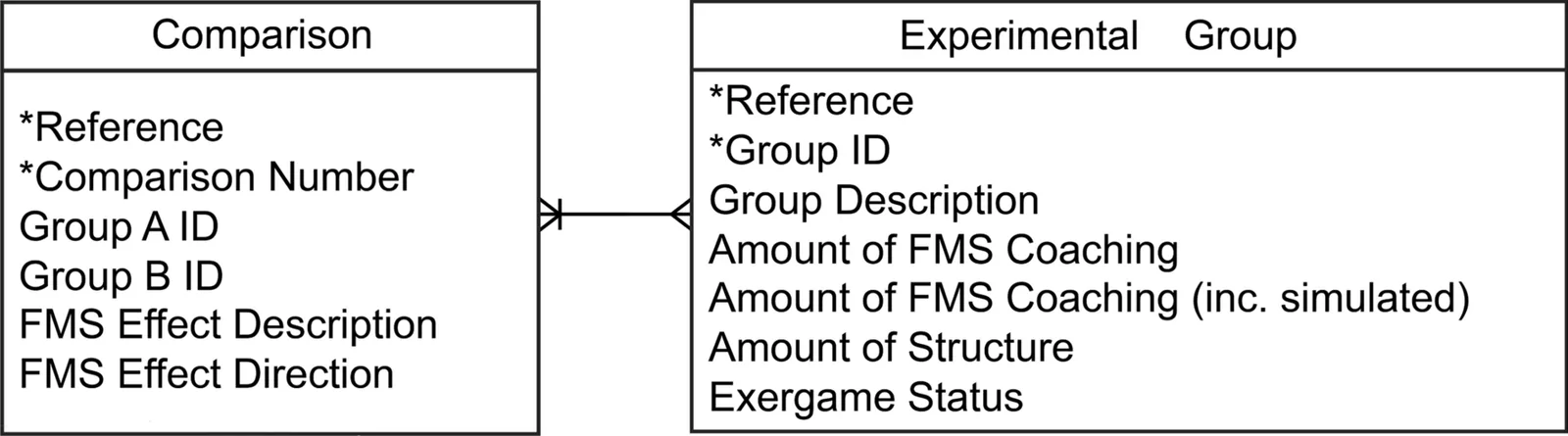
Entity-relationship diagram of the comparison and experimental group data tables. The data tables are available in full in the supplementary material. *FMS* Fundamental Movement Skills, *ID* Identifier

A dataset of experimental comparisons was produced by extracting and categorising data from the corpus. The dataset was comprised of two tables: comparisons, and experimental groups (see Fig. 4). A table of comparisons was produced, because some studies used more than two experimental groups, and had more than one pairwise comparison. The data for both tables are available in the supplementary material.

Each comparison table entry details the results of one pairwise comparison between two groups. Comparison data comprised two experimental group identifiers and an effect which is classified as either increasing FMS, decreasing FMS or having no effect on FMS. Group identifiers are referred to as being on side A or B in the comparison, based on the direction the comparison was made in the paper. For example, [23] compared two groups, which we label as CG1 and EXG1 for clarity. The group CG1 received 10 PE lessons in a 4 week span and EXG1 received 5 PE lessons and 5 sessions, where they were supervised playing exergames in a 4 week span. The results from the study[23] show that the difference between condition A (CG1) and condition B (EXG1) was a lowered rate of FMS acquisition that was statistically significant, implying that if a group was in condition A and the condition changed to B then there would be a drop in the rate of FMS acquisition. The comparison table entry for the study[23] recorded the comparison as finding a decrease in FMS, owing to the negative effect of moving from condition A to B.

The experimental group table describes what the differences were between the groups in the comparisons. Based on the results of group categorisation, nominal values such as 0 or 1 were used to represent how the methodological variable changed between groups. For example, for the study conducted by [23], the descriptions of each group were recorded as “PE lessons 5 times per two weeks" for CG1 and “Alternating exergame and PE lessons 5 times per two weeks" for EXG1 in the experimental groups table. The paper made no mention of any coaching during exergaming and described the exergaming sessions as supervised. For the amount of FMS coaching CG1 was given a nominal value of 1 and EXG1 was given a nominal value of 0, to record that in total EXG1 received less FMS coaching than CG1. For the amount of structure, CG1 was given a nominal value of 1 and EXG1 was given a nominal value of 0, because the exergame sessions were free style play. For the exergame status, CG1 was given a value of 0, because it contained no exergames, and EXG1 was given a value of 1, because it contained more exergaming time than the group it was compared with. Nominal values made it possible to compute the prediction a hypothesis would make for an experimental set up in the next stage. Nominal values were assigned by using a text value such as “PE class" which were then turned into a number by finding the matching value in a look up table. The look up table is available in the supplementary material.

### Meta-analysis of the Effects of Methodological Variables

We used a systematic hypothesis testing approach to perform our meta-analysis. We predicted results for each of the 11 hypotheses using the dataset of experimental procedures that we produced in the previous stage. Predictions were then compared with study results to determine which hypothesis was supported by the evidence. The dataset and predictions are summarised in Sects. What Methodological Variables Exist Amongst Research into the use of Exergamoids to Improve Children’s FMS? (RQ1) and Can the Variance in FMS Effects be Associated with Variances in the Methodology? (RQ2), respectively, and are available in the supplementary material.

**Fig. 5:**
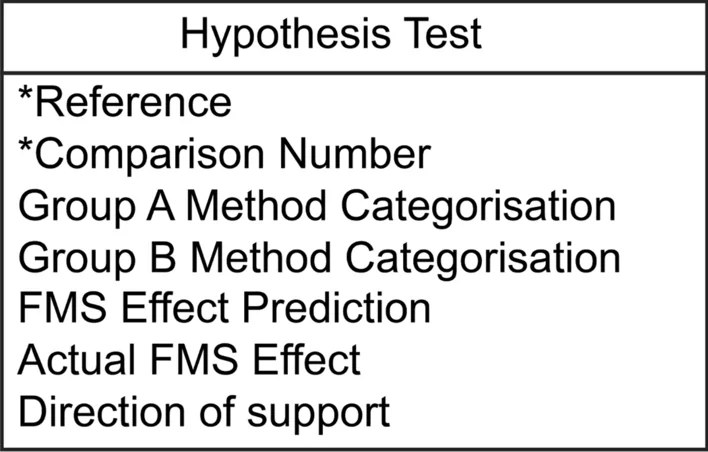
Entity-relationship diagram of the hypothesis test tables. We produced hypothesis test tables for each hypothesis by processing the extracted study data. The data are available in the supplementary material. *FMS* Fundamental Movement Skills

We processed the experimental comparisons dataset to create 11 hypothesis test tables (see Fig. 5). Each hypothesis test table explored all 17 comparisons and one hypothesis about the cause of variability in FMS effects. The hypothesis test table included the reference, experimental groups, categorised in terms of the methodological variables in the hypothesis, and the FMS effect direction.

Making a prediction for a study involved identifying what the methodology was. The methodology was determined by considering how each group in the comparison was categorised. The way that the group method was categorised was dependent on the hypothesis under test. For example, for the comparison in [23], when testing hypothesis $H_G$, which posits that only exergame status influences results, Group A Method was categorised as “Traditional Activity" and Group B Method was categorised as “Exergame Activity". Only these methodological details were used as input to compute the prediction for $H_G$, because $H_G$ posits that results can be predicted from just these variables. The hypothesis $H_C$ posits that variance was because FMS coaching improves FMS, and exergame status influenced results positively or negatively. When testing $H_C$ for the study [23], Group A Method was categorised as “Regular coaching&Traditional Activity" and Group B Method was categorised as “Supervision without FMS feedback&Exergame Activity". These methodological details were used as input to compute the prediction for $H_C$. The group method data field did not aim to describe the experimental groups in specific detail, but to identify the direction of change in the methodological variables. The experiment described by [23] effectively measures the effect of reducing coaching and increasing exergames, a movement between the category of “Regular coaching&Traditional Activity" and “Supervision without FMS feedback&Exergame Activity". The variance in each methodological variable was determined from the experimental group methodology classifications. The nominal values were used to determine the direction of each change to a methodological variable, to compute a prediction using the hypothesis under test. Where there were two variables in the set, predictions were combined, as shown in Table 2. Where there were more than two variables, predictions were combined iteratively.

If the observed effect was in the same direction as the combined prediction then the comparison was marked with a ▴ on the hypothesis test table to indicate the results support the hypothesis prediction. If the observed effect was outside of the range the comparison was marked with ▾ to show the results contradict the hypothesis prediction. If the hypothesis led to a prediction of $\Delta F \in \mathbb {R}$ then the comparison was marked with $\blacktriangleleft \blacktriangleright$ to show that conclusions about the hypothesis could not be informed by this combination of hypothesis and experimental set up. This comparison of hypothesis predicted effect and actual effect was performed for all 17 comparisons entries.

This process was repeated for all 11 hypothetical models. We manually combined these tables into the final result matrix (see Sect. Can the Variance in FMS Effects be Associated with Variances in the Methodology? (RQ2)) by compiling the support column from each table. Statistical significance was assessed to determine the best explanation for variance in FMS results.

**Table 2:** Combination matrix used to predict the combined effects on fundamental movement skills for a multifactorial intervention, based on partial predictions for factors A and B

Prediction *A*	No change $\Delta F_B\in \{0\}$	Increase $\Delta F_B\in \mathbb {R}_{>0}$	Decrease $\Delta F_B \in \mathbb {R}_{<0}$	Increase/Decrease $\Delta F_B\in \mathbb {R}_{\ne 0}$
Prediction *B*				
No change $\Delta F_A\in \{0\}$	$\Delta F_{(A,B)}\in \{0\}$	$\Delta F_{(A,B)}\in \mathbb {R}_{>0}$	$\Delta F_{(A,B)}\in \mathbb {R}_{<0}$	$\Delta F_{(A,B)}\in \mathbb {R}_{\ne 0}$
Increase $\Delta F_A\in \mathbb {R}_{>0}$	$\Delta F_{(A,B)}\in \mathbb {R}_{>0}$	$\Delta F_{(A,B)}\in \mathbb {R}_{>0}$	$\Delta F_{(A,B)} \in \mathbb {R}$	$\Delta F_{(A,B)} \in \mathbb {R}$
Decrease $\Delta F_A\in \mathbb {R}_{<0}$	$\Delta F_{(A,B)}\in \mathbb {R}_{<0}$	$\Delta F_{(A,B)} \in \mathbb {R}$	$\Delta F_{(A,B)} \in \mathbb {R}_{<0}$	$\Delta F_{(A,B)} \in \mathbb {R}$
Increase/Decrease $\Delta F_A \in \mathbb {R}_{\ne 0}$	$\Delta F_{(A,B)}\in \mathbb {R}_{\ne 0}$	$\Delta F_{(A,B)} \in \mathbb {R}$	$\Delta F_{(A,B)} \in \mathbb {R}$	$\Delta F_{(A,B)} \in \mathbb {R}$

ΔF: predicted change in fundamental movement skills. $\Delta F_{A}, \Delta F_{B}$: the predicted effects for the selected methodological variables *A* and *B*. $\Delta F_{(A,B)}$: the combined predicted effects for the selected methodological variables *A* and *B*. $\Delta F_{(A,B)} \in \mathbb {R}_{>0}$ indicates a combined prediction of a positive effect. $\Delta F_{(A,B)} \in \mathbb {R}_{<0}$ indicates a combined prediction of a negative effect. $\Delta F_{(A,B)} \in \mathbb {R}_{\ne 0}$ indicates a combined prediction of a positive or negative effect. $\Delta F_{(A,B)} \in \mathbb {R}$ indicates indeterminate effects, where a clear prediction cannot be made

## Results

### What Methodological Variables Exist Amongst Research into the use of Exergamoids to Improve Children’s FMS? (RQ1)

Across the 17 comparisons, there were experiments involving modifying a single variable, and experiments involving modifying multiple variables at the same time. The experimental designs involved combinations of the following methodological variables:

- Whether the activity was an exergame or a traditional physical activity.
- Whether readily available commercial games were used or researcher-made games with design enhancements for FMS were used.
- Whether the intervention took place as a structured PE session or a less structured supervised playtime.
- Whether the group received targetted coaching related to FMS skills alongside activities.

Table 3 shows a summary of the methodological variables that were changed in each comparison and the measured effect upon FMS. More than one methodological variable was modified in some comparisons (see Table 3). In most papers, the variance in coaching or structure was well-defined due to large differences between groups, or no differences. Where we tested hypotheses involving $C_{(H,D)}$, the coaching level was determined as the qualitative net result of human delivered $C_H$ and digital media delivered components $C_D$. There was only one paper [24], where combined effects of human and digital components were considered to determine the overall coaching level. In the paper by [24] the PE control group practised a different movement skill to the one that was measured. The intervention group lost this form of coaching input, but received skill demonstration videos as feedback which were relevant to the movement skill measured in the study. We classified the intervention group as receiving a higher level of coaching. Some researcher-made games discussed the specifics of the exergame used in the intervention [23–25]. We categorised differences between groups in finer detail where possible, depending on the level of methodological detail in the paper. If we categorised all comparisons using the coarser scale, $H_E$ would still have been the most supported hypothesis.

There were variances in methodology which were not explored as experimental variables in a comparison within the corpus: the trial length; the age range; time of day; and the FMS measurement tool used and thereby the temporal, environmental, and functional skill aspects assessed. In our meta-analysis we explored if there are differential effects of exergames upon the temporal, environmental, and functional skill aspects of FMS.

Table 4 shows the age-spans used in the comparisons. All studies used a span of ages, due to the availability of groups of participants in a school setting. The age spans cover all ages within the age-range for our meta-analysis with no gaps. Ages at the extremes of the range are less represented, as may be expected from our selection criteria excluding studies that span across our age-eligibility range. Clusters of similar age spans appear, where a study utilised more than two experimental groups to make multiple statistically significant comparisons.

There were 22 unique games, counting titles involving minigames, such as Wii Sports, as a single game (see Table 4). There were 4 different hardware platforms. Games were categorised as commercial, research, or not specified depending on the author’s indication (see Sect. Content Analysis and Classification Scheme for our classification method). Across the 17 comparisons, 4 involved the use of a researcher made game, 12 used commercial games, and 1 used a game that was not specified, but descriptions were consistent with a commercial game. Some researcher-made games had more fine-grained detail about the intervention procedures. Classifications of comparisons involving researcher-made games were made using more detail, because more detail was available in the paper. There were limited results for non-exergame exergamoids that have been studied with a post-test measure of FMS. One comparison of two different forms of digitally gamified exercise [26] was identified but not included in our meta-analysis, so we could increase the applicability of our findings by focusing upon exergames. All studies in our corpus for the meta-analysis involved the use of exergames, and other forms of exergamoid were not present.

**Table 3:** Summary of how the methodological variables were changed in each comparison and the measured effect upon FMS

Reference	ΔS	$\Delta C_H$	$\Delta C_D$	ΔE	ΔF
[27] CG1 vs EXG1^1^	No change	Inc	No change	Inc	Inc
[27] CG2 vs EXG1 ^*^	No change	No change	No change	Inc	No change
[28] CG1 vs EXG1 ^*^	No change	Inc	No change	Inc	Inc
[28] CG2 vs EXG1 ^*^	No change	No change	No change	Inc	No change
[29]	No change	No change	No change	Inc	No change
[30] CG1 vs EXG1 ^*^	Inc	Inc	No change	Inc	Inc
[30] CG1 vs CG2 ^*^	Inc	Inc	No change	No change	Inc
[31]	Inc	No change	No change	Inc	Inc
[32]	No change	No change	No change	Inc	No change
[23]	Dec	Dec	No change	Inc	Dec
[33]	No change	No change	No change	Inc	No change
[34]	No change	No change	No change	Inc	No change
[25]	No change	No change	Inc	No change	Inc
[35]	No change	Inc	No change	Inc	Inc
[24] CG1 vs EXG1 ^1^	No change	Dec	Inc	Inc	Inc
[24] CG1 vs EXG2 ^*^	No change	Dec	Inc	Inc	Inc
[24] EXG1 vs EXG2 ^*^	No change	No change	Inc	No change	Inc

* The study used more than two experimental groups, and made more than one pairwise comparison between groups. The characteristics of groups EXG1, EXG2, CG1, and CG2 are defined for each study, in Table 4

ΔS, Change in level of structure, $\Delta C_{(H,D)}$ Change in level of coaching, including through digital media, ΔE Change in the percentage of sessions using an exergame, e.g., $E = \frac{\text {number of exergaming sessions}}{\text {total sessions}}$ ΔF, Measured change in FMS performance level. Inc. = Methodological variable increased/increase in FMS observed Dec. = Methodological variable decreased/decrease in FMS observed No change = No change in methodological variable/no statistically significant difference in FMS

**Table 4:** Characteristics of included studies

Reference	Movement skills assessed	Trial length	Group ID	Age range	Sample size (Male, Female)	Activity description	Platform(s)/Video Game(s)
[27]	Single-leg and tandem stance balance assessed using the HUR BT4 platform	6 weeks, 34 min 3 days a week	CG1	6–9	21 (M=8 F=13)	Regular PE classes	N/A
			CG2	6–9	22 (M=9 F=13)	PE Classes with an enhanced agility, balance and coordination focus, with no exergames	N/A
			EXG1	6–9	22 (M=12 F=10)	Exergaming group with expert curriculum plan	Wii: Wii Fit Plus
[28]	Single-leg and tandem stance balance, assessed using the HUR BT4 platform	6 weeks, 34 min 4–5 days a week	CG1	9–10	21 (M=12 F=9)	Regular PE classes	N/A
			CG2	9–10	19 (M=11 F=8)	PE Classes with an enhanced agility, balance and coordination focus, with no exergames	N/A
			EXG1	9–10	21 (M=10 F=11)	Exergaming group with expert curriculum plan	iDance, Wii, XR-Board Dueller System, Lightspace Play Wall: inbuilt games (iDance/XR Board/Lightspace), Wii Fit Plus
[29]	TGMD-2 (object control skills)	6 weeks, 1 h 1 day per week	CG1	4–8	53 (M=21 F=32)	Regular after school/outside of school activities	N/A
			EXG1	4–8	41 (M=22 F=19)	Afterschool exergames	Wii: Wii sports resort
[30]	TGMD-2 (object control skills)	8 weeks, 30 min 2 days per week	CG1	6–7	22 (M=13 F=9)	Unstructured free play	N/A
			CG2	6–7	22 (M=11 F=11)	Traditional FMS programme	N/A
			EXG1	6–7	22 (M=12 F=10)	FMS training programme with exergames	Kinect: Kinect Sports (Kinect), NBA Baller Beats (Kinect)
[31]	Custom tests: Tapping a balloon to keep it in the air, 3 shuttle runs (2 m each)	40 min	CG1	5–6	53 (M=28 F=25)	Active learning with an equivalent traditional game, facilitated by peers	N/A
			EXG1	5–6	52 (M=28 F=24)	Active learning with an equivalent exergame	Xtion PRO: The Goalkeeper
[32]	TGMD-3 (two hand strike, one hand strike, ball bounce, catch, kick, underarm throw, overarm throw), golf swing and putt stroke	6 weeks, 50 min, 1 day per week	CG1	6–10	17 (M=9 F=8)	Normal lunchtime activities	N/A
			EXG1	6–10	19 (M=10 F=9)	Exergaming in lunchtime break	Kinect: Kinect Sports Season 1, Kinect Sports Season 2, Kinect Sports Rivals, Kinect Adventure
[23]	Custom tests: Kicking, throwing, standing long jump, hopping	9 months, 25 min 2.5 days per week (5 days in 2-week period)	CG1	7–9	115 (M=56 F=59)	PE lessons 5 times per 2 weeks	N/A
			EXG1	7–9	135 (M=65 F=70)	Alternating exergame and PE lessons 5 times per 2 weeks	Kinect, Wii: possibly others that are unspecified, Kinect Ultimate Sports, Just Dance, Wii Sports, Wii Fit
[33]	TGMD-2	8 weeks, 30 min 5 days per week	CG1	4–5	36 (M=14 F=22)	Outdoor recess time	N/A
			EXG1	4–5	20 (M=11 F=9)	Supervised exergaming play	Wii, Kinect: Just Dance for Kids (Wii), Nickelodeon Fit (Wii), Just Dance for Kids (Xbox 360)
[34]	TGMD-2	9 weeks, 45 min 2 days per week	CG1	8–10	32 (M=∼15 F=∼17) *	Regular PE classes	N/A
			EXG1	8–10	32 (M=∼15 F=∼17)*	PE class using exergames	Kinect: Kinect Sports, Kinect Sports: Season Two, Kinect Adventures
[25]	TGMD-2 (run hop jump slide only) and skip (as in TGMD-1)	8 weeks, two 60–90 s bouts 5 days per week	CG1	5–7	20 (M=11 F=9)	Short burst with commercial exergames	Kinect, Wii: Kinect sports, EA sports active, your shape: fitness evolved
			EXG1	5–7	20 (M=10 F=10)	Short burst with researcher-made adaptable FMS-targeting Exergame	Kinect via Kinect2Scratch: Hop Ball, Jump Ball, Slide Ball, Alien Attack (modified for research by author)
[35]	TGMD-3,BOT-2 - balance component	7 weeks, 1 h 3 days per week	CG1	10–12	60 (M=∼25 F=∼35) *	Regular PE classes	N/A
			EXG1	10–12	60 (M=∼25 F=∼35) *	Exergaming programme group	Kinect: Unnamed commercial Kinect minigames: Volleyball, Tennis games, Basketball games, Standing twists games, Running games, Dancing games
[24]	Static front header, diagonal running header (from F-MARC test battery)	6 weeks, 10 min per week	CG1	9–11	18 (M=∼7 F=∼11) *	Regular PE classes	N/A
			EXG1	9–11	22 (M=∼9 F=∼13) *	Header training game, with no pedagogical progression	Oculus Quest: Rezzil Player22
			EXG2	9–11	21 (M= ∼8 F= ∼13) *	Header training game, with pedagogical progression - skill videos in response to performance	Oculus Quest: Unnamed VR soccer game

*TGMD-1* Test of Gross Motor Development: 1st edition, *TGMD-2* Test of Gross Motor Development: 2nd edition, *TGMD-3* Test of Gross Motor Development: 3rd edition, *BOT-2*= Bruininks–Oseretsky Test of Motor Proficiency: 2nd edition, *F-MARC test battery* FIFA Medical Assessment and Research Centre Test Battery

^*^ The study reported gender ratios for the overall cohort only. Group-specific values were approximated using the overall cohort ratio

### Can the Variance in FMS Effects be Associated with Variances in the Methodology? (RQ2)

The 11 hypotheses in Table 5 show all plausible hypothetical explanations for variability in FMS within the corpus. We created plausible constraints, for use in hypotheses, based on our current understanding for each methodological variable:

- If coaching during an exergame intervention is a component of FMS variability, we expect a positive correlation, defined as $\quad \frac{\partial F}{\partial C} > 0$.
- If structure during an exergame intervention is a component of FMS variability, we expect a positive correlation, defined as $\quad \frac{\partial F}{\partial S} > 0$.
- If exergame status is a component of FMS variability, we expect a non-zero effect, defined as $\quad \frac{\partial F}{\partial E} \ne 0$.

Coaching could be considered a combination of human and digital media factors or just human factors. We explored both categorisations within hypothesis modelling. $C_H$ is the level of coaching, considering only human delivered components. $(C_H+C_D)$ is the combined level of coaching, considering both digital media and human delivered components. Due to the 2 categorisation options, there were 2 sets of constraints from which some or all elements could be selected to define a hypothesis (see Eq. 1):

1 $$\begin{aligned} &A = \{\quad \frac{\partial F}{\partial S}> 0,\quad \frac{\partial F}{\partial C_H }> 0,\quad \frac{\partial F}{\partial E} \ne 0\} \\&B = \{\quad \frac{\partial F}{\partial S}> 0,\quad \frac{\partial F}{\partial (C_H + C_D)} > 0,\quad \frac{\partial F}{\partial E} \ne 0\}\\&\begin{array}{l} \text {where} \\ F \text { : Fundamental movement skill performance level.} \\ S \text { : Level of structure.} \\ C_H \text { : Level of coaching through verbal feedback}\\ \quad \text { and live demonstration.} \\ C_D \text { : Level of coaching through digital media.} \\ E \text { : Amount of activity in the form of an exergame.} \end{array} \end{aligned} $$

All possible hypotheses were defined by combining the powersets of the plausible constraints under the two variable categorisation schemes, excluding the empty set (see Eq. 2). A descriptive overview of each hypothesis that was defined is available in Table 6, and the mathematical definitions of each hypothesis are available in the supplementary material:

2 $$\begin{aligned} M = (\mathcal {P}(A)\cup \mathcal {P}(B))-\emptyset \\ F_N \text { is a function with the constraints } M_N\\ H_{A...K}: F_{1...11}\\ \begin{array}{l} \text {where} \\ \text { A and B are defined as shown in Eq. 1} \\ \end{array} \end{aligned}$$

The direction of effects table (see Table 5) show the degree of support each hypothesis has from the available evidence. Some papers have multiple entries within Table 5, because they used more than two experimental groups and made multiple statistically significant comparisons. Some predictions were not possible, where exergame element having a non-zero effect was used as part of the hypothesis. Some studies show similar patterns of hypothesis support in Table 5 due to similarity of experimental set up (see Table 3).

The hypothesis $H_G$ asserts that exergames have an effect upon FMS, and the mix of evidence that support and refute this hypothesis illustrate the mixed findings within the field. The high *p* value for $H_G$ makes this explanation unlikely. The hypotheses with most support ($H_D, H_E, H_I, H_J$) share a common element of hypothesising that human delivered coaching increases FMS and that exergame status has zero effect on FMS. The hypotheses $H_D$ and $H_E$ differ in whether they propose that coaching through digital media improves FMS. $H_E$ asserts that coaching through digital media enhances FMS, while $H_D$ posits no such effect. $H_D$ is refuted by four comparisons across two studies [24, 25], demonstrating that coaching through digital media improves FMS. The hypotheses $H_J$ and $H_E$ differ in whether they propose that structured activities improve FMS. $H_E$ asserts that structured activities improve FMS, while $H_J$ posits no such effect. $H_J$ was refuted by one study [31], indicating structure may have a role in increasing FMS within the corpus, but further research is needed.

Overall, the hypothesis that has the strongest support is $H_E$. All available statistically significant evidence supports this hypothesis, so this is the most likely explanation for variance within the corpus. The evidence is not congruent with any other hypothetical combination of methodological variables (see Table 5). Given the results for $H_E$ the effect of exergame status upon results is in the corpus zero.

**Table 5:** Direction of effect plot showing the level of support for each hypothesis amongst comparisons in the corpus

Reference	$H_A$	$H_B$	$H_C$	$H_D$	$H_E$	$H_F$	$H_G$	$H_H$	$H_I$	$H_J$	$H_K$
[27] CG1 vs EXG1 ^*^	$\blacktriangleleft \blacktriangleright$	▴	$\blacktriangleleft \blacktriangleright$	▴	▴	$\blacktriangleleft \blacktriangleright$	▴	▾	▴	▴	$\blacktriangleleft \blacktriangleright$
[27] CG2 vs EXG1 ^*^	▾	▾	▾	▴	▴	▾	▾	▴	▴	▴	▾
[28] CG1 vs EXG1 ^*^	$\blacktriangleleft \blacktriangleright$	▴	$\blacktriangleleft \blacktriangleright$	▴	▴	$\blacktriangleleft \blacktriangleright$	▴	▾	▴	▴	$\blacktriangleleft \blacktriangleright$
[28] CG2 vs EXG1 ^*^	▾	▾	▾	▴	▴	▾	▾	▴	▴	▴	▾
[29]	▾	▾	▾	▴	▴	▾	▾	▴	▴	▴	▾
[30] CG1 vs EXG1 ^*^	$\blacktriangleleft \blacktriangleright$	$\blacktriangleleft \blacktriangleright$	$\blacktriangleleft \blacktriangleright$	▴	▴	$\blacktriangleleft \blacktriangleright$	▴	▴	▴	▴	$\blacktriangleleft \blacktriangleright$
[30] CG1 vs CG2 ^*^	▴	▴	▴	▴	▴	▴	▾	▴	▴	▴	▴
[31]	$\blacktriangleleft \blacktriangleright$	$\blacktriangleleft \blacktriangleright$	▴	▴	▴	▴	▴	▴	▾	▾	$\blacktriangleleft \blacktriangleright$
[32]	▾	▾	▾	▴	▴	▾	▾	▴	▴	▴	▾
[23]	$\blacktriangleleft \blacktriangleright$	$\blacktriangleleft \blacktriangleright$	$\blacktriangleleft \blacktriangleright$	▴	▴	$\blacktriangleleft \blacktriangleright$	▴	▴	▴	▴	$\blacktriangleleft \blacktriangleright$
[33]	▾	▾	▾	▴	▴	▾	▾	▴	▴	▴	▾
[34]	▾	▾	▾	▴	▴	▾	▾	▴	▴	▾	▾
[25]	▾	▾	▾	▾	▴	▴	▾	▾	▾	▴	▴
[35]	$\blacktriangleleft \blacktriangleright$	▴	$\blacktriangleleft \blacktriangleright$	▴	▴	$\blacktriangleleft \blacktriangleright$	▴	▾	▴	▴	$\blacktriangleleft \blacktriangleright$
[24] CG1 vs EXG1 ^*^	$\blacktriangleleft \blacktriangleright$	▴	$\blacktriangleleft \blacktriangleright$	▾	▴	$\blacktriangleleft \blacktriangleright$	▴	▾	▾	▴	$\blacktriangleleft \blacktriangleright$
[24] CG1 vs EXG2 ^*^	$\blacktriangleleft \blacktriangleright$	▴	$\blacktriangleleft \blacktriangleright$	▾	▴	$\blacktriangleleft \blacktriangleright$	▴	▾	▾	▴	$\blacktriangleleft \blacktriangleright$
[24] EXG1 vs EXG2 ^*^	▾	▾	▾	▾	▴	▴	▾	▾	▾	▴	▴
Sign test: one-tail *p* value	1.00	0.79	0.99	0.02	p<0.001	0.83	0.69	0.31	0.072	p<0.001	0.91

* The study used more than two experimental groups, and made more than one pairwise comparison between groups. The characteristics of groups EXG1, EXG2, CG1, and CG2 are defined for each study, in Table 4

$H_{A...K}$ are described in Table 6 and mathematically defined in the supplementary material. ▴, Results from this comparison within the corpus support the hypothesis, ▾ Results from this comparison within the corpus contradict the hypothesis $\blacktriangleleft \blacktriangleright$, Predictions would not be possible for this combination of hypothesis and experimental set up

**Table 6:** Components of each potential hypothesis for the cause of variability in FMS results within the corpus

Hypothesis for the cause of variability in FMS results within the corpus	Hypothesis components				
	Structure improved FMS	Coaching through verbal feedback and live demonstration improved FMS	Coaching through pre-made digital media improved FMS	Exergames had a positive or negative effect on FMS	No other factors had an effect on FMS
$H_A$	✓	✓		✓	✓
$H_B$	✓			✓	✓
$H_C$		✓		✓	✓
$H_D$	✓	✓			✓
$H_E$	✓	✓	✓		✓
$H_F$		✓	✓	✓	✓
$H_G$				✓	✓
$H_H$	✓				✓
$H_I$		✓			✓
$H_J$	✓	✓			✓
$H_K$	✓	✓	✓	✓	✓

$H_{A...K}$ are described by this table, and are defined mathematically in the supplementary material. *FMS* Fundamental Movement Skills

### What Type of Effect does Each Methodological Variable Have on FMS? (RQ3)

The *p* values for the single-variable hypotheses ($H_G, H_H, H_I, H_J$) indicate the influence of each methodological variable upon variability of FMS results within the corpus. The hypothesis $H_J$ posits that coaching, including through digital media, improves FMS. The *p* value for hypothesis $H_J$ in contrast to other single-variable hypotheses indicates that coaching, including through digital media, had a strong effect upon variability of FMS effects within the corpus. Human delivered coaching during exergame play improves FMS $\quad \frac{\partial F}{\partial C_H} > 0$. There is some evidence that structured sessions involving exergames improve FMS $\quad \frac{\partial F}{\partial S} > 0$. Structure alone is not a statistically significant predictor of FMS results in the corpus as seen in $H_H, p = 0.31$. This may be because more studies explored changes in the coaching level and exergame status. Hypotheses $H_J$ and $H_E$ differ in whether they propose that the level of structure has an FMS effect. The higher level of support for $H_E$ indicates that structure is a component of the factors that have FMS effects. Switching between exergames and traditional activities has no effect on FMS$\quad \frac{\partial F}{\partial E} = 0$. The absence of exergame status as a component of the most supported model indicates exergame status has no effect. The high *p* value for $H_G$, combined with a large amount of studies altering exergame status also indicates there is no influence from this variable. Columns ΔF and ΔE of Table 3 illustrate that there is no clear link between exergame status *E* and observed FMS effects *F*.

Based on the available evidence the most supported model of effects upon FMS is $H_E$,p<0.001. Table 7 shows the methodological variables in the papers, filtered by the most supported hypothesis, $H_E$. The table illustrates a clear link between structure (column ΔS) coaching (column $\Delta C_{(H,D)}$) and observed FMS effects (column ΔF). Exergame status is not included in Table 7, because this $H_E$ predicts the effect of this factor on FMS to be 0. The measured change in FMS is listed in column ΔF. Table 7 shows that all studies support $H_E$. The measured change in fundamental movement skill performance can be predicted by combining the change in level of structure and change in level of coaching using Table 2. Because column ΔF in Table 7 can be predicted without considering changes in exergame status ΔE in the comparisons, this demonstrates that switching from traditional activity to exergame, or vice versa, had no FMS effect. Single variable comparisons of exergames and traditional activity, combined with coaching at a controlled level [27, 28, 34] measured no difference in FMS (see Table 7). Indicating there is no statistically significant differential coefficient for exergames that modulates the effectiveness of coaching.

**Table 7:** Overview of methodological variables and ordinal results in the corpus, displaying only the methodological variables predicted as explaining variability in FMS by $H_E$, and the measured change in FMS

Reference	ΔS	$\Delta C_{(H,D)}$	ΔF
[27] CG1 vs EXG1 ^*^	No change	Increase	Increase
[27] CG2 vs EXG1 ^*^	No change	No change	No change
[28] CG1 vs EXG1 ^*^	No change	Increase	Increase
[28] CG2 vs EXG1 ^*^	No change	No change	No change
[29]	No change	No change	No change
[30] CG1 vs EXG1 ^*^	Increase	Increase	Increase
[30] CG1 vs CG2 ^*^	Increase	Increase	Increase
[31]	Increase	No change	Increase
[32]	No change	No change	No change
[23]	Decrease	Decrease	Decrease
[33]	No change	No change	No change
[34]	No change	No change	No change
[25]	No change	Increase	Increase
[35]	No change	Increase	Increase
[24] CG1 vs EXG1 ^*^	No change	Increase	Increase
[24] CG1 vs EXG2 ^*^	No change	Increase	Increase
[24] EXG1 vs EXG2 ^*^	No change	Increase	Increase

* The study used more than two experimental groups, and made more than one pairwise comparison between groups. The characteristics of groups EXG1, EXG2, CG1, and CG2 are defined for each study, in Table 4

The measured change in fundamental movement skill performance can be predicted by combining the change in level of structure and change in level of coaching using Table 2. Changing between traditional activity and exergame has no influence on results. ΔS, Change in level of structure, $\Delta C_{(H,D)}$, Change in level of coaching, including through digital media, ΔF, Measured change in FMS performance level. Increase= Increase in methodological variable/statistically significant increase in FMS observed Decrease = Decrease in methodological variable/statistically significant decrease in FMS observed No change = No change in methodological variable/no statistically significant difference in FMS

Table 8 shows the one sided sign test *p* values for each hypothesis using only the subset of our corpus related to each skill component. Skills assessments that were used in the corpus typically involved closed skills in assessments. There were no fully open skills but a subset of the corpus involved slightly open skills, for example, catching a ball that is being thrown to the center of the body. For studies that involved stability skills as part of assessment, the supported hypotheses are $H_D H_E H_I H_J$. Hypotheses $H_D H_E H_I H_J$ predict exergames are not a factor influencing FMS variability in the corpus. We consider the evidence for stability skills to be that human-delivered coaching improves FMS, exergame status has no effect, and there is not enough information to determine the role of digitally delivered coaching or the role of structure for stability skills. $H_E$, is statistically significant across almost all skill components. Across all skill components there is no statistically significant evidence for exergames having a differential effect due to the skill components assessed in the FMS measure.

**Table 8:** Analysis of differential effects for methodological variables across the temporal, environmental, and functional skill aspects of FMS

Skill component	$H_A$	$H_B$	$H_C$	$H_D$	$H_E$	$H_F$	$H_G$	$H_H$	$H_I$	$H_J$	$H_K$
Closed	1.00	0.89	0.98	0.002	p<**0**.**001**	0.91	0.87	0.13	0.01	0.002	0.96
Open*	0.98	0.75	0.98	0.17	p<**0**.**001**	0.89	0.83	0.38	0.17	p< **0**.**001**	0.89
Stability	1.00	0.50	1.00	**0**.**03**	**0.03**	1.00	0.50	0.81	**0**.**03**	**0**.**03**	1.00
Manipulative	0.98	0.75	0.94	0.07	p<**0**.**001**	0.77	0.61	0.19	0.19	0.003	0.89
Locomotor	1.00	0.94	0.94	0.11	**0.02**	0.69	0.66	0.34	0.34	0.11	0.88
Continuous	1.00	0.77	1.00	0.06	**0**.**008**	0.94	0.77	0.77	0.06	**0**.**008**	0.94
Discrete	0.98	0.75	0.98	0.27	p< **0**.**001**	0.66	0.73	0.50	0.27	p< **0**.**001**	0.66
Serial	1.00	0.88	0.88	**0.03**	**0.03**	0.88	0.50	0.19	0.19	0.19	1.00

$H_{A...K}$ are described in Table 6 and mathematically defined in the supplementary material. The most supported hypothetical model for each skill component is highlighted in bold. p<0.05 = Results for this skill component show statistically significant support for the hypothesis. *Open skills typically only involved a timing element

## Discussion

### Low Barrier Exergames

Previous research into the effects of exergames on FMS has reported mixed conclusions. Our meta-analysis controls for methodological variables. Every experimental result in the corpus can be explained by the hypothesis that improvements of FMS are a function of coaching and activity structure, and that exergames are just as effective as traditional physical activities for improving FMS. The hypothesis $H_E$ predicted all the experimental results seen in the corpus, and is defined mathematically in equation (3). No other hypothesis could predict all experimental results. This is a surprising finding as it is possible to operate many of the exergames that were used in the studies with different upper limb kinematics, which would suggest an impaired skill transfer [36].

The findings of our meta-analysis are consistent with the dynamic systems theory (DST) of motor skills acquisition. DST is a set of mathematical concepts that have been applied to many disciplines [37]. DST describes how complex patterns could emerge over time from many smaller systems self-organising by following simple rules [37]. When applied to motor skills acquisition it theorises how motor skills may originate from the biological systems of the body [37]. FMS acquisition can be thought of as a search for a correct movement pattern by coordinating movements across many degrees of freedom. The mathematics of dynamic systems theory would indicate that a system could stabilise at different speeds, or even not stabilise at all, depending on the characteristics of feedback. Characteristics of the feedback include how effectively it narrows the search space, and how it guides away from local maxima created in other feedback schemes. Another key theoretical point for DST in the context of motor skills acquisition, is that there needs to be emotional significance to the feedback from the movement to motivate change [37]. In childhood, emotionally significant movement feedback might be scoring points, or receiving praise. The search to acquire FMS could be informed by knowledge of results (KR) or knowledge of performance (KP). In exergames and traditional activities, KR may originate from the game scoring and intrinsic feedback from the simulation or real world activity. KP might be provided through coaching by the facilitator. In this phase of childhood, KR would not provide clear information about how to alter movements to improve FMS. KP from adult facilitators would provide actionable information about how to refine movements. Indicating why FMS acquisition is most strongly influenced by interaction with mentors, rather than the accuracy of the simulation. The interchangeability of exergames and traditional activity may not translate to other forms of motor learning, where KP can be more easily connected with movement adaptations, or where the learning goal is outcome related (e.g., improving throw accuracy in a traditional setting):

3 $$\begin{aligned} &H_E:F = f(S, C_H+C_D), \quad \frac{\partial F}{\partial S}> 0, \\ &\quad \frac{\partial F}{\partial (C_H+C_D)} > 0, \quad \frac{\partial F}{\partial E} = 0 \\& \begin{array}{l} \text {where} \\ F \text { : Fundamental movement skill performance level.} \\ S \text { : Level of structure.} \\ C_H \text { : Level of coaching through verbal feedback} \\ \quad \text { and live demonstration.} \\ C_D \text { : Level of coaching through digital media.} \\ E \text { : Amount of activity in the form of an exergame.} \end{array} \end{aligned}$$

One aspect of structure and coaching was selecting activities that aligned with developmental needs, as seen in 6 papers in our corpus [25, 27, 28, 30, 34, 35]. We note that exergames with permissive sensors and inaccuracies in simulation are equally as effective for motor development as traditional activities, which are inherently physically accurate. This pattern demonstrates that the gross motor specificity requirements set by the equipment for an FMS activity in childhood can be a superset of the desired skill, rather than the exact skill. For example, to train a jump, the game needs only respond to a correct jump, it does not need to reject or give a lower reward for an incorrect jump. Through the nature of FMS coaching, facilitators will already be co-creating the specificity of the activity sufficiently through the social dynamic of coaching. In effect, facilitators increase specificity by setting performance goals for additional praise. The specificity of an activity involves the intersection of facilitator and equipment-driven constraints.

The findings of our meta-analysis present an alternate explanation to those in [18], which reported a mildly positive FMS effect of exergames overall, and speculated these were driven by enhanced specificity in balance skills. Table 8 shows that the most supported hypothesis from Table 5, $H_E$, is statistically significant across almost all skill components. Column $H_G$ of Table 8 shows that there is no statistically significant relationship between exergames and stability skills, or any other skill component. The studies driving the positive effect in [18] either have no significant effect when controlled for methodological variables [27, 28, 30], or included a non-typical population, such as developmentally delayed children with conditions affecting balance [38]. This indicates that coaching and structure is a more congruent explanation for the positive effects seen in some exergame studies, and confirms that exergames are just as effective as traditional activities for skills development for all skill types.

Our meta-analysis shows a pattern of unstructured activities being less effective at developing FMS and a possible explanation for the pattern in [39]. The preschool age-range, which showed structured activities may have a greater motor skill effect than unstructured activities, was unable to demonstrate a statistically significant relationship due to methodological variability. Our meta-analysis indicates that the methodological variability within structured activities in this age range might also be due to variance in the level of focus on FMS training. In our corpus, structure sometimes included planned opportunities for FMS coaching or activity selection informed by FMS considerations. This indicates the presence of a skill mentor during unstructured activities may be needed to help interpret the significance of unstructured discoveries. Exergames providing more structure to the activity will not be enough to drive a motor skills effect. Exergames will be more effective in less-structured play through inclusion of FMS coaching elements.

The broader goal of FMS within promoting health is to develop a series of baseline skills that can be specialised in a later developmental stage. Skills enable access to sports, which in turn involve exercise. Low-barrier exergames in childhood can disrupt the inactivity cycle in ways that traditional PE cannot. Exergames can be used to develop movement skills that may not have been practical in a school setting before, such as surfing and snowboarding between classes. In addition to skills development, learning the game rules, and becoming familiar with the sights and sounds may act as a form of exposure therapy. [24] Notable were decreased fears around heading a ball, when children trained heading skills in virtual reality. Exergames can enable effective training during periods of bad weather. Exergames can promote exertion through heart-rate trackers [40, 41]. Thus, exergame awareness provides a back up route to physical activity, where advanced movement skills are not needed. There are indications that exergames may have an enhanced effect in the groups that traditional activity does not reach, such as developmentally delayed children [38] and children who do not attend PE sessions regularly [42]. We propose that including a mix of exergames and traditional physical activities within childhood curricula leads to reduced barriers for active lifestyles throughout life.

Schools may need to overcome several challenges to implement exergames in PE. The cost of purchasing exergames may be a limiting factor [27, 28, 30], so additional funding may be needed. PE staff may need training on how to select appropriate games, how to adapt gameplay based on needs, and the importance of human-delivered feedback and instruction as noted in this paper. Key considerations when selecting a game, that have been raised in the corpus include: if movements align with the scheme of work [34], if the game operates by the full correct movement skill [24], what the activity timings are [25], the level of support for classroom management needs [23], and capacity for differentiation [25]. Games that are not designed for the purpose of being run in a PE session can have challenges in customising the game for different ability levels and session timings [25]. Behaviour management plans should also consider if ’cheating’ within the chosen game gives better scores than playing with the correct movement. Incentives to cheat, opposed to a skill neutral scoring response, may increase the difficulty of monitoring progress in groups with challenging behaviour. School IT infrastructure may need additional technical support capacity, though children are often able to resolve issues involving setting up and playing games [27, 28, 30].

As game developers create games that more accurately sense motor skills, the low-barrier reward framework may be more effective in engaging children in physical activity than strict simulations that pose a skill barrier. The ‘cheatability’ of low-barrier exergames enables children’s moral engagement with agreed rules, similar to sport. [29] Found that most children preferred competitive games, where it was easy for them to succeed. Permissive sensors that offer a low barrier to activity might be an ideal arrangement for FMS coaching, because the teacher can adjust the threshold for praise to match the needs of the individual child. A high skill barrier introduces competing negative feedback from overly challenging physical factors of the task, e.g. improvements in swing technique but still missing a baseball. For low-barrier feedback to be effective we would recommend game developers design games, where ’cheating’ gives a score that is no better than playing with the correct movement. We consider low-barrier exergames to be a distinct category of exergames due to the potential for lasting value and unique deployment considerations.

### Simulated Coaching

Our meta-analysis indicates there are different types of exergames with respect to FMS effects. Exergames with coaching through digital media improve FMS, whereas exergames without these elements do not have any difference with traditional activities. The methods coaches used to turn low-barrier exergames into FMS learning opportunities indicate how exergames can be enhanced to independently increase FMS. Human coaches improved FMS within low-barrier exergame studies by selecting and adapting activities for training needs, modelling skills, and cueing. We hypothesise that exergames can have an enhanced FMS effect by combining coaching techniques to simulate coaching interactions more fully. Exergames can involve feedback skill demonstrations [24] which have a similar mechanism to cueing and demonstration. Exergames can be used to assess general movement competence [43], which can form part of selecting activities based on need or cueing. Considering that KP is a more actionable form of feedback than KR for FMS adaptation, future work should explore if sensor improvements can effectively improve FMS through improvements in simulated coaching, rather than improvements in simulated game worlds. As noted with human coaching of exergames, an option for a low barrier approach to game world simulation can help with onboarding. We consider simulated coaching exergames to be a distinct category of exergames that is worthy of further research.

### Co-teaching Systems

To inform future research, it must be determined if simulated coaching is expected to produce consistent effects in settings, where there is already a teacher, by acting as a teaching assistant (TA). There are varying perceptions of the TA role and a need for clarity in the strategies TAs should employ [44]. The lack of clarity makes it difficult to determine if a game can employ the cooperative education strategies a TA does. Based on TA literature, a simulated coach working effectively alongside a teacher would involve more than being able to teach FMS skills. A key cooperative challenge with TAs is that they can be counter productive by displacing interaction with the teacher [45]. A co-teaching game would need to avoid displacing teacher interaction by adapting around the teacher’s activities. For example, in [27] games were selected by the teacher based on FMS needs, rather than activity choices being driven by the game. The FMS needs would have been determined by the teacher’s assessments and previous activities. In [25] the teacher controlled the intensity of activities based on fitness assessments. A teacher may also prefer to customise game-delivered instructional elements in response to previous achievement and learning objectives. Co-teaching games require an additional layer of interactivity, so that the teacher can plan and choose the learning activities that game delivers. The unique customisability requirements establish co-teaching games as a different subcategory of exergames. Although exergames have not demonstrated an ability to teach on the level of a teacher they could still be useful. Collaborative teaching has been shown to still yield benefits when the skill level of the second collaborative teacher is not as high [46]. The potential for co-teaching indicates that future exergames that meet certain criteria may be more effective activities for FMS teaching than traditional activities.

Our meta-analysis shows that low-barrier exergames are best thought of as activities that can be coached, rather than skill-delivery systems. Low-barrier exergames are just as effective as a traditional activity in a coaching experience targeting FMS. The emerging evidence indicates this finding could change with future iterations of the technology. Developments in modelling skills to enhance performance, delivering accurate cues that constitute knowledge of performance, sensing exertion, and coordinating with teachers seem likely to have enhanced effectiveness in certain settings. Low-barrier exergames and game modes will still have applications, so the findings of this study will remain relevant. We recommend using the classification of exergamoid types to both organise information and help generalise findings in a future-oriented way.

## Conclusion

Our meta-analysis shows that the key variable causing mixed FMS results in exergame research is the amount of adult facilitation. This methodological variable explains all of the variance in group-level results in exergame studies. Controlling for facilitation, it emerges that exergame activities are just as effective as traditional activity for FMS development; and that ’cheatable’ games are not damaging to FMS adaptation when not facilitated. The implications are exergames should be freely chosen by teachers, where they see them aligning with FMS needs. Parents can immediately make use of exergames in the home environment, by encouraging their child to “play properly" by coaching them on the proper version of the movement and moving vigorously. Low-barrier exergames should continue to be produced by developers. The ‘cheatability’ of low-barrier games is desirable, because it enables children with imperfect technique to participate and may be providing a motivational benefit.

Our meta-analysis focused on a whole class level and did not explore the influence of individual participant characteristics on the effectiveness of exergames or traditional activities, for which we anticipate an effect. There was also no evidence available to determine if small differences arise or not over interventions that last longer than 6–8 weeks. These are areas which could be covered in future research. Remaining gaps in the literature that warrant further investigation are how exergamoids can best model skills, sense skill performance, deliver cues, adapt to exertion ability, and coordinate with teachers. Future research should build on our mapping of statistically significant effects in order to manage the methodological variability that makes building on previous findings challenging.

In conclusion, low-barrier exergames are just as effective as traditional activity for FMS adaptation. Expanding the range of options for children with exergamoids will help overcome the practical challenges that are spiralling into physically inactive lifestyles.

## Additional file

Supplementary file 1.

Supplementary file 2.

Supplementary file 3.

Supplementary file 4.

Supplementary file 5.

Supplementary file 6.

Supplementary file 7.

Supplementary file 8.

Supplementary file 9.

Supplementary file 10.

Supplementary file 11.

Supplementary file 12.

Supplementary file 13.

Supplementary file 14.

Supplementary file 15.

Supplementary file 16.

Supplementary file 17.

Supplementary file 18.

Supplementary file 19.

Supplementary file 20.

## Data Availability

All data generated or analysed during this study are included in this published article and its supplementary information files.

## Abbreviations

FMS: Fundamental Movement Skills
KP: Knowledge of Performance
KR: Knowledge of Results
PA: Physical Activity
PE: Physical Education
PES: Physical Education and School sport
PICO: Population, Intervention, Comparison, Outcome
TA: Teaching Assistant

## Footnotes

1 The systematic review management platform used in this study was Rayyan.
