# Supplementary material for: The Effects of Exergames on Fundamental Movement Skills in Childhood: A Systematic Review and Meta-analysis
Source: Sports Med Open. 2026 Sep 20;12:139. doi: 10.1186/s40798-026-01081-2 (PMC13590462; doi:10.1186/s40798-026-01081-2)
Supplement: Supplementary file 1 [file 40798_2026_1081_MOESM18_ESM.pdf]

1 Hypothesis definitions

- H_A : Coaching and activity structure increase FMS, exergames have either a positive or negative effect on FMS, there are no other factors
  $F = f(S, C_H, E), \quad \frac{\partial F}{\partial S} > 0, \quad \frac{\partial F}{\partial C_H} > 0, \quad \frac{\partial F}{\partial E} \neq 0$
- H_B : Structure increases FMS, exergames have either a positive or negative effect on FMS, there are no other factors.
  $F = f(S, E), \quad \frac{\partial F}{\partial S} > 0, \quad \frac{\partial F}{\partial E} \neq 0$
- H_C : Coaching increases FMS , exergames have either a positive or negative effect on FMS, and there are no other factors.
  $F = f(C_H, E), \quad \frac{\partial F}{\partial C_H} > 0, \quad \frac{\partial F}{\partial E} \neq 0$
- H_D : Coaching and activity structure increase FMS, and there are no other factors.
  $F = f(S, C_H), \quad \frac{\partial F}{\partial S} > 0, \quad \frac{\partial F}{\partial C_H} > 0,$
- H_E : Coaching (inc. digital media) and activity structure increase FMS, and there are no other factors.
  $F = f(S, C_H + C_D), \quad \frac{\partial F}{\partial S} > 0, \quad \frac{\partial F}{\partial (C_H + C_D)} > 0$
- H_F : Coaching (inc. digital media) increases FMS , exergames have either a positive or negative effect on FMS, and there are no other factors.
  $F = f(C_H + C_D, E), \quad \frac{\partial F}{\partial (C_H + C_D)} > 0, \quad \frac{\partial F}{\partial E} \neq 0$
- H_G : Exergames have a positive or negative FMS effect and there are no other factors.
  $F = f(E), \quad \frac{\partial F}{\partial E} \neq 0$
- H_H : Structured activities increase FMS, and there are no other factors.
  $F = f(S), \quad \frac{\partial F}{\partial (S)} > 0,$
- H_I : Coaching increases FMS, and there are no other factors.
  $F = f(C_H), \quad \frac{\partial F}{\partial (C_H)} > 0,$
- H_J : Coaching (inc. digital media) increase FMS, and there are no other factors.
  $F = f(C_H + C_D), \quad \frac{\partial F}{\partial (C_H + C_D)} > 0,$

- H_K : Coaching (inc. digital media) and activity structure increase FMS, exergames have either a positive or negative effect on FMS, there are no other factors

  $F = f(S, C_H + C_D, E), \quad \frac{\partial F}{\partial S} > 0, \quad \frac{\partial F}{\partial (C_H + C_D)} > 0, \quad \frac{\partial F}{\partial E} \neq 0$

where

F : Fundamental movement skill performance level.

S : Level of structure.

C_H : Level of coaching through verbal feedback and live demonstration.

C_D : Level of coaching through digital media.

E : Amount of activity in the form of an exergame.
